# Xuebijing Injection Promotes M2 Polarization of Macrophages and Improves Survival Rate in Septic Mice

**DOI:** 10.1155/2015/352642

**Published:** 2015-05-10

**Authors:** Yan-Cun Liu, Feng-Hua Yao, Yan-Fen Chai, Ning Dong, Zhi-Yong Sheng, Yong-Ming Yao

**Affiliations:** ^1^Department of Emergency Medicine, Tianjin Medical University General Hospital, Tianjin 300052, China; ^2^Trauma Research Center, First Hospital Affiliated to the Chinese PLA General Hospital, Beijing 100048, China; ^3^State Key Laboratory of Kidney Disease, The Chinese PLA General Hospital, Beijing 100853, China

## Abstract

Xuebijing (XBJ) injection, a concoction of several Chinese herbs, has been widely used as an immunomodulator for the treatment of severe sepsis in China. However, the precise mechanisms responsible for its efficacy have not been fully elucidated. In our study, we determined the flow cytometry markers (F4/80, CD11c, and CD206), the levels of secreted cytokines (TNF-*α*, IL-6, and IL-10), and the expression of specific proteins of M2 (Ym1, Fizz1, and Arg1) to assess macrophage polarization. Treatment with XBJ lowered M1 associated cytokine levels and increased the level of M2 associated cytokine level. The percentage of M2 phenotype cells of XBJ group was much higher than that of the control group. Expressions of phosphorylated Janus kinase 1 (JAK1) and signal transducer and activator of transcription 6 (STAT6) were markedly enhanced after the administration of XBJ; on the other hand, the M2 associated cytokines and proteins were decreased following treatment with JAK1 or STAT6 inhibitor. In addition, the treatment of XBJ significantly improved the survival rate of septic mice. These studies demonstrate that XBJ can markedly promote M2 polarization and improve the survival rate of septic mice, thereby contributing to therapeutic effect in the treatment of septic complications.

## 1. Introduction

Sepsis, a clinical syndrome occurring in patients after infection secondary to acute insults, is the leading cause of mortality of patients in ICU worldwide [1]. It is estimated that there are 750,000 cases of sepsis in the United States, which cost 17 billion dollars each year, and the number continues to climb year by year [2, 3]. Although it has been demonstrated that there are complex reactions of inflammation, immune dysregulation, and coagulation dysfunction in the pathogenesis of sepsis [4], the precise pathogenic mechanisms remain to be further elucidated.

Macrophages are involved in the process of the innate immune response and play a significant role in sepsis and other inflammatory diseases [5, 6]. In the early stage of sepsis, macrophages secrete a large number of inflammatory mediators which incite the cytokine storm and septic shock which is the main cause of death in severe sepsis [7]. Macrophages display remarkable plasticity, and they can give rise to different populations of cells with distinct functions in response to different environmental conditions [8, 9]. In this polarization spectrum, M1 (classically activated macrophage) and M2 (alternatively activated macrophage) represent the two poles. M1 shows a proinflammatory character, which secretes tumor necrosis factor- (TNF-) *α*, interleukin- (IL-) 6, and other cytokines, which induce the uncontrolled inflammation and even septic shock, while M2 possesses an anti-inflammatory character, which secretes IL-10 and other cytokines and reduces the overstimulated inflammation. Researches have shown that the amount of IL-10 and the ratio of M1/M2 are prognostic markers of survival in septic experiments in gorilla [10]. Therefore, tuning the polarization of macrophage and regulating the balance of macrophage hold great promise in the treatment of septic complications [7].

XBJ is a complex traditional prescription of herbs which is extracted from* Paeonia lactiflora (Chi Shao), Ligusticum chuanxiong (Chuan Xiong), Codonopsis pilosula (Dan Shen), Carthamus tinctorius (Hong Hua)*, and* Angelica sinensis (Dang Gui)*, and early study from HPLC-ESI-MS has identified that it consists of paeoniflorin (1962.99 mg/L), senkyunolide I (53.15 mg/L), safflor yellow A (48.66 mg/L), danshensu (48.33 mg/L), ferulic acid (37.40 mg/L), and twenty-one other compounds [11, 12]. It has been widely used in China in recent years as an anti-inflammatory drug for the treatment of sepsis and various inflammatory diseases, and it shows a promising clinical therapeutic effect [13, 14]. Though studies have shown that XBJ reduces the septic syndrome by inhibiting nuclear factor- (NF-) *κ*B signal pathway [15] and promotes annexin A1 expression and inhibits proinflammatory cytokines secretion [16], the precise mechanism of its therapeutic effect of this complex compound calls for further evaluation.

We hypothesized that XBJ could affect the phenotype of macrophage favoring a M2 state that promoted the resolution of inflammatory reaction. In the current study, we found that XBJ markedly promoted the polarization of macrophage in sepsis and thus improved the survival rate of septic mice induced by cecal ligation and puncture (CLP), and it might be a potential mechanism affording the protective effect of XBJ on sepsis.

## 2. Materials and Methods

### 2.1. Chemicals and Reagents

XBJ was obtained from Tianjin Chase Sun Pharmaceutical Co. Ltd. (Tianjin, China) with the lot number of 1204181. Thioglycollate broth, lipopolysaccharide (LPS), and leflunomide were purchased from Sigma-Aldrich (St. Louis, MO). Allophycocyanin- (APC-) anti-CD11c and fluorescein isothiocyanate- (FITC-) anti-CD206 were purchased from BioLegend (San Diego, CA). Phycoerythrin- (PE-) anti-F4/80 was purchased from eBioscience (San Diego, CA). Mouse TNF-*α*, IL-6, and IL-10 enzyme-linked immunosorbent assay (ELISA) kits were purchased from Excel Bio company (Shanghai, China). JAK1 and STAT6 fast activated cell-based ELISA (FACE) kits were purchased from Active Motif (Carlsbad, CA). Mouse anti-Arg1 was purchased from BD Biosciences (San Jose, CA), mouse Fizz1 antibody was purchased from R&D Systems (Minneapolis, Minn), anti-Ym1 was purchased from Stemcell Technologies (Vancouver, Canada), and anti-*β*-actin was purchased from Santa Cruz Biotechnology (Santa Cruz, CA). JAK1 inhibitor was purchased from Calbiochem (San Diego, CA).

### 2.2. The Extraction of XBJ

Roots of* Paeonia lactiflora (Chi Shao)*, roots of* Ligusticum chuanxiong (Chuan Xiong)*, roots of* Codonopsis pilosula (Dan Shen)*, flowers of* Carthamus tinctorius (Hong Hua)*, and roots of* Angelica sinensis (Dang Gui)* were dried, pulverized, and extracted using methanol-water in an ultrasonic water bath. The near-infrared spectroscopy (NIRS) was used to determine the major constituents online to ensure the consistence and stability of XBJ. Its major constituents included paeoniflorin (1962.99 mg/L), senkyunolide I (53.15 mg/L), safflor yellow A (48.66 mg/L), danshensu (48.33 mg/L), ferulic acid (37.40 mg/L), and twenty-one other compounds [11, 12].

### 2.3. Mice

Male Balb/c mice (6–8 weeks old, 18–22 g) were obtained from Laboratory Animal Center of Chinese Academy of Medical Sciences, Beijing. They were housed in separate cages in a temperature-controlled room with 12 h light/dark artificial light cycle to acclimatize for at least 7 days before being used. All experimental protocols were carried out conforming to the National Institute of Health Guide for the Care and Use of Laboratory Animals, with the approval of the Scientific Investigation Board of the Chinese PLA General Hospital, Beijing, China.

### 2.4. Cell Culture

Primary peritoneal macrophages were isolated from Balb/c mice (male, 6–8 weeks old, 18–22 g) 2-3 days after receiving intraperitoneal injection of 2 mL thioglycolate broth (4%) as previously described [17], with minor modifications. In brief, peritoneal cavity was washed with phosphate-buffered saline (PBS), and cells were grown in Dulbecco's modified Eagle's medium (DMEM) containing 10% fetal bovine serum (FBS) in 5% CO_2_ at 37°C for 2 h. Adherent macrophages were washed with PBS twice and cultured in fresh DMEM medium containing 10% FBS.

### 2.5. CLP Induced Sepsis

An experimental sepsis model was reproduced by CLP, as previously described [18]. In brief, after a satisfactory anesthesia, a 1.5 cm midline incision was made, and the cecum was exposed and ligated at the point distal to the ileocecal valve. The cecum was punctured with 21G needle in the midline and squeezed to extrude about 2 mm of fecal content. Then, the cecum was returned to its normal position, the abdominal incision was closed, and prewarmed normal saline was injected to resuscitate animals. Thereafter, XBJ (4 mL/kg) or normal saline was intramuscularly administered at 0.5 h, 12 h, 24 h, 36 h, 48 h, 60 h, 72 h, 84 h, and 96 h after CLP. Seven-day survival rate was observed. At 24 h after CLP, peritoneal macrophages were collected for phenotypic analysis with flow cytometry, and blood samples were collected for measurement of serum concentrations of TNF-*α*, IL-6, and IL-10 with ELISA kits.

### 2.6. Flow Cytometry

Cultured macrophages and peritoneal macrophages isolated after CLP were harvested and washed with cold PBS twice. Collective cells were, respectively, stained with PE-anti-F4/80, APC-anti-CD11c, and FITC-anti-CD206 for 30 min at 4–8°C in darkness and washed twice by centrifugation in FACS buffer, and then they were fixed with 1% paraformaldehyde. Cells were analyzed on a FACSCalibur. Flow cytometric data were analyzed using the CellQuest software (Becton Dickinson Immunocytometry Systems, San Jose, CA).

### 2.7. Western Blotting

Proteins from whole cell lysates of macrophages were separated on 4%–10% Tris-glycine gels and transferred to nitrocellulose membranes. Membranes were probed with primary antibodies and then incubated with a secondary antibody conjugated with horseradish peroxidase. All immunoblots were visualized with enhanced chemiluminescence. The protein levels were quantitated with densitometry using Bio-Rad Quantity One Software.

### 2.8. Expression of JAK1 and STAT6

Expressions of total and active (phosphorylated) JAK1 and STAT6 were quantified using fast activated cell-based ELISA assays strictly according to the manufacturer's instructions (Active Motif, Carlsbad, CA).

### 2.9. Cytokine Measurement

TNF-*α*, IL-6, and IL-10 levels were determined with ELISA kits, strictly following the procedures provided by the manufacturers. The color reaction was terminated by adding 100 *μ*L of orthophosphoric acid. Plates were read in a microplate reader (Spectra MR, Dynex, USA).

### 2.10. CCK-8 Proliferation

Cell viability was assessed by the CCK-8 strictly following the protocols provided by the manufacturer. Briefly, peritoneal macrophages were plated into 96-well flat bottom plates at 1 × 10^5^ cells/well and cultured. After treatment with different doses of XBJ, 10 *μ*L CCK-8 solution was added to each well in the plate and the absorbance was read in a microplate reader (Spectra MR, Dynex, USA) at OD 450 nm.

### 2.11. Statistical Analysis

Statistical analysis was performed using SPSS software (Version 13). All results were presented as mean ± SEM. One-way ANOVA analysis and Student's *t*-test were used to assess the differences between various experimental and control groups. Values of *p* < 0.05 were accepted as significant. Differences in survival rate were analyzed by a Kaplan-Meier survival plot and log-rank statistics.

## 3. Results

### 3.1. Moderate Dose of XBJ Enhanced Viability of Macrophages

**Figure 1:**
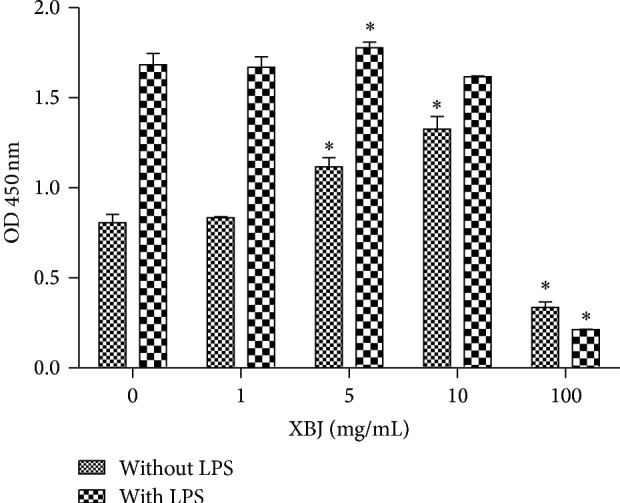
Moderate dose (5 mg/mL) of XBJ enhanced viability of macrophages. Mouse peritoneal macrophages were treated with 0 mg/mL, 1 mg/mL, 5 mg/mL, 10 mg/mL, and 100 mg/mL XBJ with or without 100 ng/mL LPS for 24 h. CCK-8 was used to assess cell viability. Data represented the mean ± SEM of independent experiment in triplicate. ^∗^ *p* < 0.01 versus control group (0 mg/mL XBJ).

Mouse peritoneal macrophages were elicited by thioglycollate broth and collected and purified by an adherence method. Viability of peritoneal macrophages was examined and verified. XBJ was added to the cell culture in different doses for 24 h with or without LPS, and cell viability was determined with CCK-8. A harmful effect to the cell viability of macrophages was noticed when the dose was higher than 10 mg/mL XBJ, and, on the other hand, the dose of 5 mg/mL XBJ showed a promoting effect of cell viability (Figure 1).

### 3.2. Moderate Dose of XBJ Promoted Transformation of Macrophages from M1 to M2* In Vitro*

**Figure 2:**
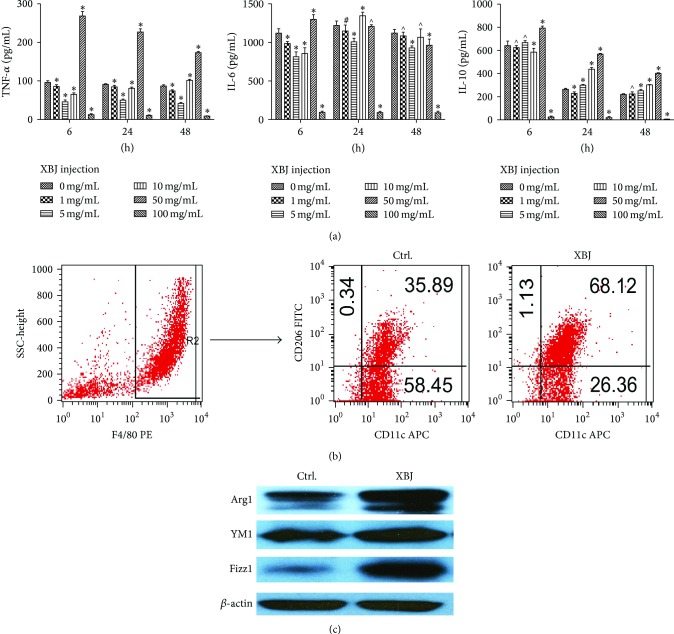
Moderate dose of XBJ (5 mg/mL) enhanced the expression of M2 phenotype markers of mouse peritoneal macrophages. Peritoneal macrophages of mice were, respectively, treated with 0 mg/mL, 1 mg/mL, 5 mg/mL, 10 mg/mL, 50 mg/mL, and 100 mg/mL XBJ in the presence of 100 ng/mL LPS for 6 h, 24 h, or 48 h. Cytokines levels including TNF-*α*, IL-6, and IL-10 in the medium were determined by ELISA (a). F4/80, CD11c, and CD206 expressions were determined by flow cytometry. The group of F4/80^+^CD11c^+^CD206^−^  was classified as M1 and F4/80^+^CD11c^−^CD206^+^ as M2 (b). The treated macrophages were lysed and levels of Arg1, Ym1, and Fizz1 were measured by western blot (c). Data represented the mean ± SEM of independent experiment in triplicate. ^∗^ *p* < 0.01, ^#^ *p* < 0.05, and ^∧^ *p* > 0.05 versus control group.

XBJ (5 mg/mL) was added to the cell culture after the stimulation of LPS for 24 h; then cell culture was collected and cells were lysed for western blotting or they were dyed using fluorescent antibodies for flow cytometric analysis. With the treatment of XBJ, M1 associated cytokine levels (TNF-*α* and IL-6) were sharply lowered, while M2 associated cytokine level (IL-10) was elevated (Figure 2(a)). The percentage of M2 phenotype (F4/80^+^CD11c^−^CD206^+^) [19] cells of XBJ group was much higher than that of the control group by flow cytometric analysis (Figure 2(b)). In addition, the effect of XBJ on the expression of Ym1, Arg1, and Fizz1, which are known to be the specific proteins of M2 polarization [20], was investigated, and results from western blotting showed that the expression of these proteins was significantly higher than that of the control group (Figure 2(c)). Therefore, it was found that XBJ could promote mouse peritoneal macrophage polarization from M1 to M2 phenotype after the stimulation of LPS* in vitro*.

### 3.3. JAK1-STAT6 Signal Pathway Was Involved in the Polarization of Macrophages Treated with XBJ

**Figure 3:**
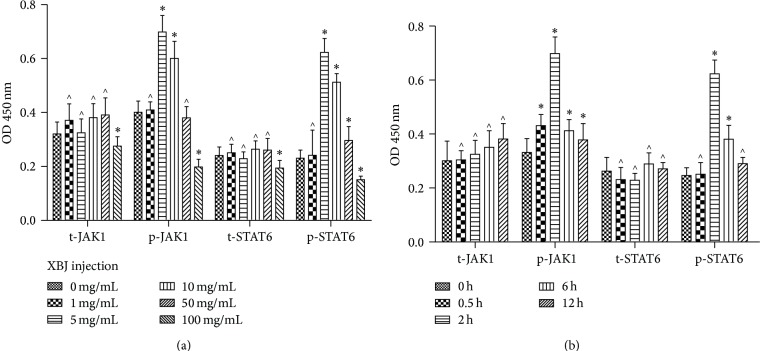
Moderate dose of XBJ (5 mg/mL) upregulated the phosphorylation of JAK1-STAT6 of mouse peritoneal macrophages. Mouse peritoneal macrophages were, respectively, treated with 0 mg/mL, 1 mg/mL, 5 mg/mL, 10 mg/mL, 50 mg/mL, or 100 mg/mL XBJ in the presence of 100 ng/mL LPS for 0 h, 0.5 h, 2 h, 6 h, and 24 h. Expressions of t-JAK1, p-JAK1, t-STAT6, and p-STAT6 were determined by fast activated cell-based ELISA. Data represented the mean ± SEM of independent experiment in triplicate. ^∗^ *p* < 0.01 and ^∧^ *P* > 0.05 versus control group.

**Figure 4:**
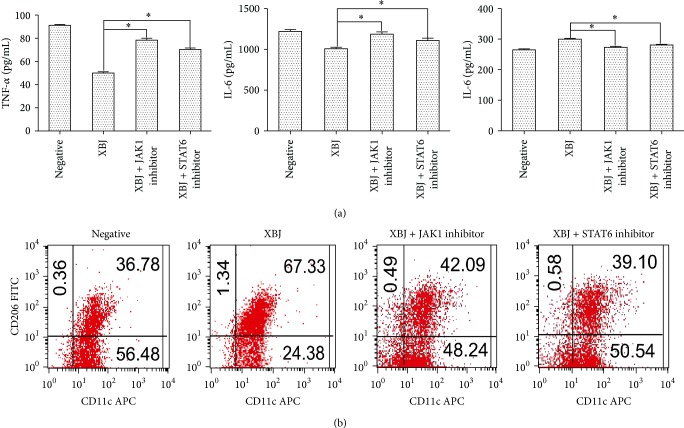
Treatment with JAK1 and STAT6 inhibitors could markedly diminish the polarization of macrophages by XBJ. Mouse peritoneal macrophages were, respectively, treated with 0 mg/mL or 5 mg/mL XBJ in the presence of 100 ng/mL LPS with or without JAK1 inhibitor or STAT6 inhibitor (leflunomide) for 24 h. TNF-*α*, IL-6, and IL-10 levels in the medium were assessed by ELISA (a). F4/80, CD11c, and CD206 expressions were analyzed by flow cytometry. The group of F4/80^+^CD11c^+^CD206^−^  was classified as M1 and F4/80^+^CD11c^−^CD206^+^ as M2 (b). Data represented the mean ± SEM of independent experiment in triplicate. ^∗^ *p* < 0.01 versus control group.

XBJ was added to the cell culture after the stimulation of LPS; then macrophages were fixed and tJAK1, pJAK1, tSTAT6, and pSTAT6 were measured at various time points. Results with ELISA showed that pJAK1 and pSTAT6 were markedly enhanced after the treatment with XBJ (Figure 3). Then, the JAK1-STAT6 signal pathway was downregulated by JAK1 [21] and STAT6 inhibitors (leflunomide) [22], and macrophage phenotypes as well as cytokines were determined with flow cytometry and ELISA, respectively. It was found that the effect of XBJ in producing macrophage polarization was obviously diminished in the presence of JAK1 or STAT6 inhibitor (Figure 4). The findings indicated that the polarization of macrophage as a result of XBJ treatment was closely related to JAK1-STAT6 signal pathway.

### 3.4. XBJ Promoted M2 Polarization* In Vivo* and Improved Survival Rate in Septic Mice

**Figure 5:**
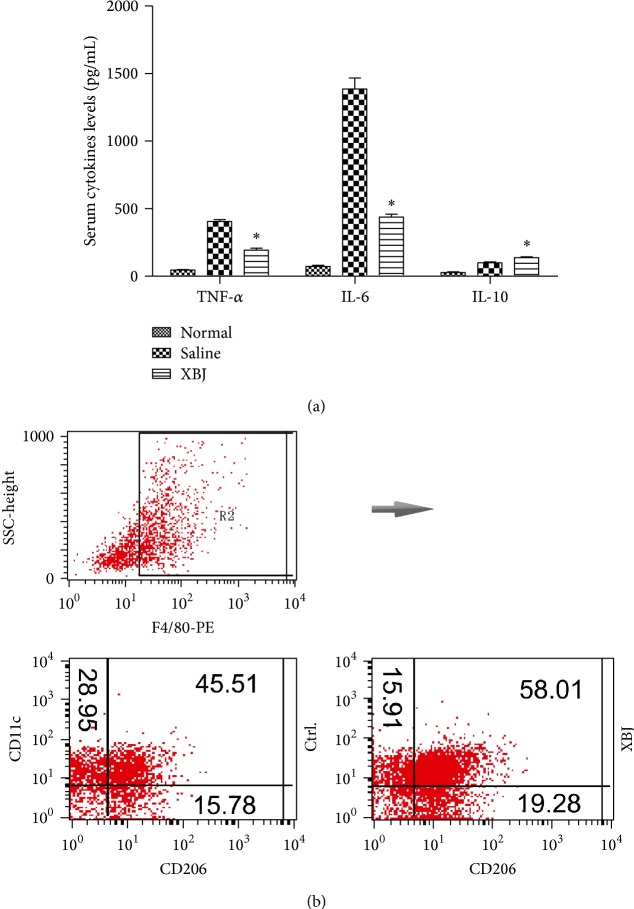
XBJ promoted the polarization of mouse peritoneal macrophages in septic mice* in vivo*. Balb/c mice were subjected to severe sepsis induced by CLP, and XBJ (4 mg/kg) was intramuscularly administered at 0.5 h and 12 h after CLP. Serum levels of TNF-*α*, IL-6, and IL-10 were determined by ELISA at 24 h after CLP (a). Peritoneal macrophages were collected and F4/80, CD11c, and CD206 expressions were analyzed by flow cytometry at 24 h after CLP. The group of F4/80^+^CD11c^+^CD206^−^  was classified as M1 and F4/80^+^CD11c^−^CD206^+^ as M2 (b). Data represented the mean ± SEM of three independent experiments. ^∗^ *p* < 0.01 versus control group.

**Figure 6:**
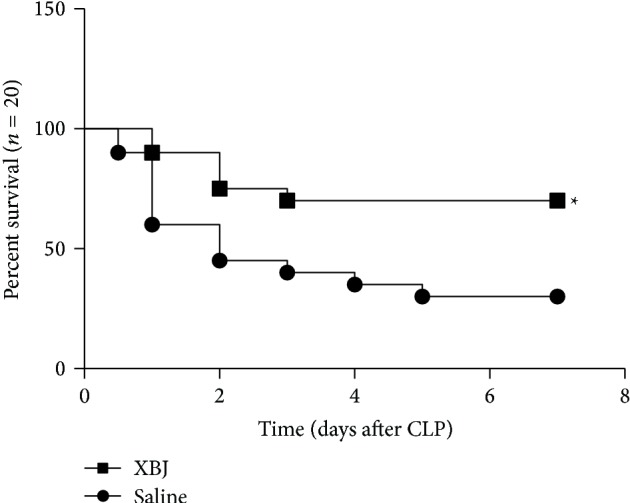
XBJ significantly improved the survival rate of septic mice induced by CLP. Balb/c mice were subjected to lethal sepsis induced by CLP, and either XBJ (4 mg/kg) or normal saline was intramuscularly administered at 0.5 h, 12 h, 24 h, 36 h, 48 h, 60 h, 72 h, 84 h, and 96 h after CLP, respectively. The differences in mortality rate between groups were compared using the Kaplan-Meier method. ^∗^Different from saline, *P* < 0.05.

Balb/c mice were subjected to severe sepsis induced by CLP, and XBJ (4 mg/kg) or normal saline was intramuscularly administered at 0.5 h and 12 h after CLP. Mice were sacrificed 24 h after CLP, and serum cytokines were measured with ELISA (Figure 5(a)), and peritoneal macrophages were collected and analyzed by flow cytometry (Figure 5(b)). The results showed that treatment with XBJ could promote the peritoneal macrophage polarization* in vivo*; thus it might downregulate the excessive inflammatory reaction and improve the survival rate of mice subjected to severe sepsis. Then we carried out the experiment for survival assessment in mice following septic challenge. Balb/c mice were subjected to lethal sepsis induced by CLP, and either XBJ (4 mg/kg) or normal saline was intramuscularly administered at 0.5 h, 12 h, 24 h, 36 h, 48 h, 60 h, 72 h, 84 h, and 96 h after CLP. It was noticed that the survival rate of XBJ group was significantly higher than that of the saline group (Figure 6).

## 4. Discussion

Sepsis is a distinct systemic response induced by occult or apparent infection, which results in inflammatory imbalance, immune dysregulation, and coagulation dysfunction [4]. With a high morbidity and mortality, it has been the leading cause of death in the ICU, and it remains as the chief medical expenses in USA. Macrophages, with the characters of diversity and plasticity, are involved in the pathogenesis of many inflammatory diseases including diabetes mellitus, asthma, and sepsis, and the regulation of macrophage polarization could be a potential strategy for its treatment [7]. In the development of sepsis, macrophage polarization is involved in the course of cytokine storm and the immune depression, which are the two main causes of death [23, 24], and regulation of the macrophage phenotypes might be considered as an effective treatment for the ailment [10]. XBJ, a traditional Chinese herbal concoction, has been widely used in China for the treatment of sepsis as well as other inflammatory diseases, and it has shown a good therapeutic effect in clinical settings. For instance, XBJ was proved as an effective measure in disseminated intravascular coagulation induced by severe sepsis, and it improved the short-term prognosis of septic patients [13]. XBJ could also alleviate renal injury in systemic lupus erythematosus-prone mice through influencing T-cell polarization mediated by dendritic cells [14]. Additionally, XBJ inhibited secretory function of Kupffer cells and attenuated liver injury in heat stroke rats [25]. In the current study, we first identified the regulatory function of XBJ in shifting the polarization of peritoneal macrophage of septic mice and its effect in improving the survival rate resulted from severe sepsis.

Our results showed that XBJ had a wide therapeutic window in the treatment of severe sepsis; however, it was found to be harmful when the dose was exceeding 10 mg/mL XBJ, which was 100 times its therapeutic dose. At the beginning, we used mouse peritoneal macrophages elicited from thioglycollate broth to conduct* in vitro* experiments, and the results demonstrated that treatment with XBJ could promote polarization of mouse peritoneal macrophage from M1 to M2 phenotype under the participation of JAK1-STAT6 signal pathway. Then, we prepared the septic model of mice by CLP, which has been recognized as the gold standard model of polymicrobial sepsis [26], and it was found that XBJ could markedly enhance M2 polarization of peritoneal macrophage* in vivo *and significantly improve the outcome of septic mice.

Diversity and plasticity were two hallmarks of macrophages, which were polarized to the exact phenotypes with their surrounding environments, and M1 as well as M2 represented the two poles of the wide spectrum. Although there is no consensus on the definite criterion for classification of M1 and M2 [9], a number of investigators have proved that the flow cytometry markers (F4/80, CD11c, and CD206) [19, 27], the secreted cytokines, and the expression of specific proteins of M2 (Ym1, Arg1, and Fizz1) [20, 28] represent the three indices to verify polarization of M2 macrophage. In the present study, it was shown that when macrophages were treated with XBJ, the flow cytometry markers of macrophages were changed from M1 to M2 phenotypes, and the expression of M2 related proteins was sharply increased; furthermore, the cytokines secreted were polarized to M2 phenotype. These findings suggested that mouse peritoneal macrophage was shifted to M2 phenotype after the treatment with XBJ, and it diminished the M1 related excessive inflammatory response and enhanced the release of anti-inflammatory cytokines. It is our belief that macrophage polarization mediated by XBJ might be closely related to the beneficial therapeutic effect in mice suffering from severe sepsis. The experimental result was consistent with a report from CLP rats which identified XBJ as an NF-*κ*B inhibitor in the treatment of sepsis [15].

JAK-STAT pathway is a widely expressed signal process, which is critically involved in the proliferation, polarization, apoptosis, and the immune regulation during various inflammatory diseases [29]. Certain previous studies revealed that JAK1-STAT6 signal pathway was a significant signal pathway underlying M2 macrophage polarization as it was involved in the expression of M2 related molecules, like Arg1, CD206, Fizz1, and Ym1 [29]. In the present study, expressions of phosphorylated JAK1 and STAT6 were markedly enhanced with the stimulation of XBJ; however, the M2 macrophage associated cytokines and proteins after being treated with XBJ were obviously decreased following treatment with JAK1 or STAT6 inhibitor. Therefore, it can be concluded that the polarization of macrophage mediated by XBJ appears to be associated with JAK1-STAT6 signal pathway. Taken together, as an activator of JAK1-STAT6 signal pathway, XBJ may be of potential benefit in the treatment of metabolic diseases and other inflammatory diseases.

## 5. Conclusions

In summary, it was evident that XBJ significantly induced the shift of macrophage from M1 to M2 phenotype, which could be a potential mechanism to explain its protective effect against sepsis, and the shift might be related to the regulation of JAK1-STAT6 signal pathway. A growing body of circumstantial evidence suggests that sepsis is a complex syndrome involving inflammation, immune imbalance, and dysregulated coagulation. The failure of different experimental therapies for sepsis in recent clinical trials [30, 31] suggests that targeting a specific signal pathway or molecule is probably not going to be effective in clinical settings. Chinese traditional medicine XBJ has been shown, including our study, to have multiple therapeutic targets [13, 15, 32, 33] in severe sepsis with diverse components [11], and it shows promise as a treatment of septic complications.
